# Activation of the cGMP/protein kinase G system in breast cancer by the dopamine receptor-1

**DOI:** 10.20517/cdr.2019.83

**Published:** 2019-12-19

**Authors:** Nira Ben-Jonathan, Dana C. Borcherding, Sejal Fox, Eric R. Hugo

**Affiliations:** Department of Cancer Biology, University of Cincinnati, Cincinnati, OH 45267, USA.

**Keywords:** Dopamine receptor-1, cGMP, protein kinase G, phosphodiesterase 5, xenografts, breast cancer

## Abstract

Despite recent advances in the detection and treatment of breast cancer, many shortcomings remain, providing incentives to search for new therapeutic targets. This review provides information on the expression and actions of dopamine receptor-1 (D1R) in breast cancer. D1R is overexpressed in a significant number of primary breast tumors, characterized by having an aggressive phenotype and predicting a shorter survival time for patients. Activation of D1R in breast cancer cells by selective agonists caused suppression of cell viability, stimulation of apoptosis, inhibition of cell invasion, and an increase in chemosensitivity. Instead of being linked to the cAMP/PKA system as expected, D1R in breast cancer is linked to the activation of the cGMP/protein kinase G (PKG) pathway. Fenoldopam, a peripheral D1R agonist that does not penetrate the brain, dramatically suppressed the growth of breast cancer xenografts in immune-deficient mice. A new imaging system for detecting D1R-expressing tumors and metastases was also developed. The review offers a novel concept that D1R can serve as a biomarker for prognosis in advanced breast cancer and its agonists can be used as effective and personalized therapeutics in a subpopulation of patients with D1R-expressing breast tumors. Several drugs, some of which are FDA-approved, that bypass the D1R and directly activate the cGMP/PKG apoptotic system, are also identified.

## Introduction

Each year, over a million women worldwide are diagnosed with breast cancer (BC). Although targeted therapies against estrogen receptors (ER) and Her2/neu have improved response rate and survival, patients with advanced disease, who are resistant to anti-hormonal therapy and/or chemotherapy, have very limited treatment options for reducing morbidity and mortality. These shortcomings provide major incentives to develop new, effective, and personalized therapies. After discovering expression of functional dopamine receptors (DARs) in human adipocytes and breast adipose tissue^[1]^, we asked whether the DARs are also expressed in BC, and if so, what are their functions and how do they act. Following analysis of 750 primary breast carcinomas, we found overexpression of dopamine receptor-1 (D1R) in ~30% of the tumors^[2]^. D1R was also overexpressed in multiple breast cancer cell lines (BCC), where D1R activation caused apoptosis and increased chemosensitivity. Unexpectedly, instead of stimulating cAMP, specific D1R agonists increased intracellular cGMP levels. Furthermore, direct activators of soluble guanylate cyclase (sGC), stimulators of protein kinase G (PKG), and selected inhibitors of phosphodiesterase 5 (PDE5), which prevents cGMP degradation, also suppressed the viability of BCC.

The objectives of this review are to provide information on the characterization of the cGMP/PKG system in BC and present data on the multiple effects and mechanism of action of various regulators of this pathways. Several drugs that intervene with this pathway and could be used as future therapeutics for combating this cancer are also identified.

## Peripheral dopamine and DARs

Dopamine (DA) is a member of the catecholamine family, which is comprised of biogenic amines with a catechol ring structure. The family includes three members: DA, norepinephrine, also known as noradrenaline, and epinephrine, also known as adrenaline. The three catecholamines share a biosynthetic pathway and have a very similar chemical structure. Despite their similarities, each of the catecholamines has a distinct tissue distribution, binds to discrete receptors, and has a different spectrum of actions.

DA acts as a major neurotransmitter in the brain and as a circulating hormone in the periphery. Circulating DA does not originate from the brain, but comes from sympathetic nerve endings, the adrenal medulla, and the GI tract^[3]^. Unique to humans, DA circulates primarily as dopamine sulfate (DA-S), a biologically inactive metabolite with a long half life of several hours, as compared with only few minutes for native DA. Many peripheral organs, including several cancers, produce arylsulfatase A (ARSA), a secretable lysosomal enzyme that can de-conjugate DA-S and convert it into bioactive DA^[4]^. Presumably, circulating DA-S serves as a stable reservoir of inactive DA that can be readily converted to authentic DA when the need arises.

**Figure 1:**
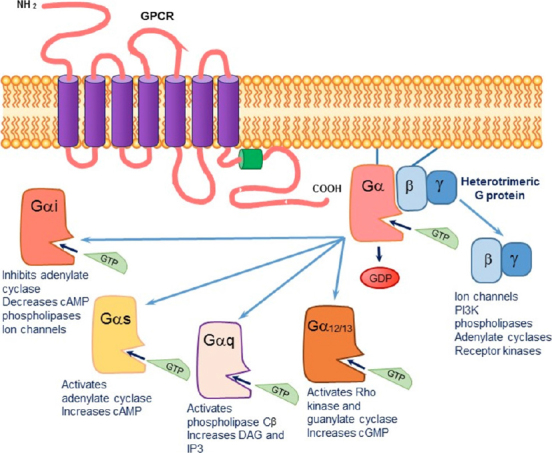
The signaling cascade initiated upon agonist binding to a G protein-coupled transmembrane receptor (GPCR). The exchange of GDP by GTP causes a dissociation of the Gα subunit from the βγ subunit. The activated alpha subunit can alter downstream effectors. Each of the alpha subunits has a specific set of second messengers, while the dissociated βγ subunit can independently activate a variety of effectors

DA binds to five G protein-coupled transmembrane receptors (GPCRs), a superfamily of receptors which lack an intrinsic kinase activity and mediate their signal transduction through the heterotrimeric G-proteins^[5]^. As illustrated in Figure 1, the GPCRs assume a seven-transmembrane α-helical configuration, predicted to form three extracellular loops and three intracellular loops (ICL). The transmembrane loops are flanked by an extracellular N-terminus and an intracellular C-terminus.

The G proteins consist of three functional subunits: α, β and γ. By some estimates, there are 15 α-subunits, 5 β-subunits, and 14 γ-subunits^[6]^. The α-subunits are grouped by sequence and functional similarities into the following: (1) Gαs (activation of adenylate cyclase; AC); (2) Gαi/olf (inhibition of certain AC isoforms); (3) Gαq (activation of phospholipase C-β); (4) Gα12 (activation of membrane Rho kinase); and (5) Gα13 (activation of guanylate cyclase; GC). Each α-subunit contains a guanine nucleotide binding site. When inactive, the α-subunit is bound to GDP and the βγ-complex, together forming a trimeric protein complex that is bound to the interface between the third ICL and the C-terminus of the receptor.

Binding of an agonist to a GPCR activates the receptor, enabling it to act as a guanine nucleotide exchanged factor. This involves the release of GDP from the α-subunit, followed by GTP binding to the α-subunit at nanomolar affinity. The exchange of GDP for GTP induces a rapid dissociation of the α-subunit from the βγ-complex, resulting in its activation [Figure 1]. Both the activated α-subunit and the freed βγ-subunit can then interact with intracellular effectors such as enzymes, transporters, or ion channels. Upon GTP hydrolysis, the GDP-bound α-subunit and the βγ-subunit re-associate into an inactive trimeric G protein, and the transmission activity of the receptor subsides.

## Canonical signaling pathways of the DARs

**Figure 2:**
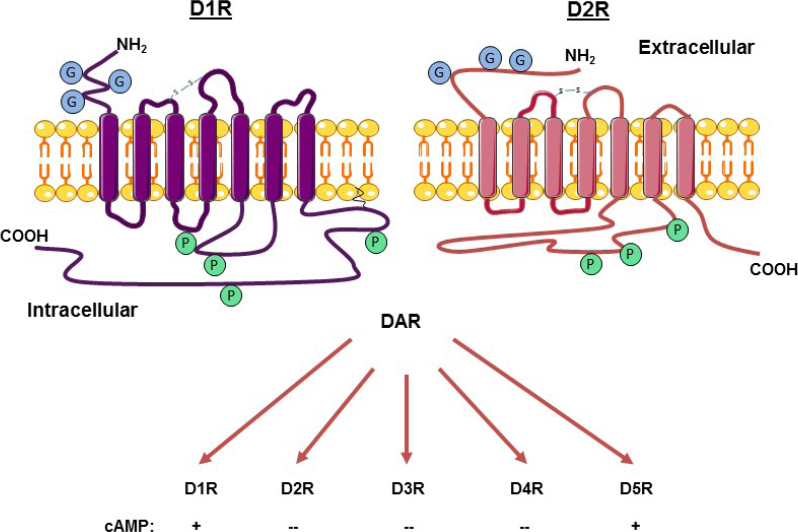
Diagram of the structure of dopamine receptor-1 (D1R) and D2R and classification by their effects on cAMP. D1-like have a longer C terminal tail and a smaller third intracellular loop that links to G-protein than D2-like

The five DARs are grouped by structure, pharmacology and function into D1-like receptors (D1R and D5R), and D2-like receptors (D2R, D3R and D4R). According to the original classification, formulated in the 1970s^[7]^, D1-like receptors are coupled to Gαs proteins, activate AC, increase cAMP, and stimulate protein kinase A (PKA), while D2-like receptors are coupled to Gαi/o proteins, inhibit AC, suppress cAMP, and inhibit PKA. Figure 2 depicts the conventional classification of the DARs by their effects on cAMP. Although this classification still holds today, the signaling cascades which are activated by the various DARs are much more variable and complex, as discussed below.

The DARs are localized in certain brain regions, where they regulate voluntary movements, reward, sleep pattern, working memory, cognitive functions, attention, feeding, olfaction, vision, and reproductive behavior^[8]^. In the periphery, DARs are expressed in multiple organs, including kidney, gut, coronary arteries, adipose tissue, reproductive organs, and immune cells, and are involved in several aspects of the general operation of the sympathetic nervous system (SNS). Peripheral DARs also participate, often independent of the SNS, in the control of cardiovascular, renal, gastrointestinal, immune, and reproductive functions. The DARs are also associated with malignancies of several peripheral organs and tissues^[9]^.

Downstream effectors of the DARs include cAMP response element binding protein (CREB), a transcription factor with multiple actions, and DARPP-32 (a 32-kDa DA- and cAMP-regulated phosphoprotein), which has protein phosphatase 1 inhibitory functions. DARPP and its truncated form, tDARPP, are over-expressed in breast, colon, esophageal, gastric, lung and prostate cancers, where they exert variable oncogenic actions^[10]^. The DARs also modulate voltage-gated K^+^_v_, Na^+^_v_, and Ca^2+^_v_ channels, while the dissociated Gβγ subunit modulates G-protein-gated, inwardly-rectifying K^+^ and Ca^2+^_v_ channels^[11]^. As described in greater detail below, D1R can also couple to other G proteins and activate alternative signaling pathways such as the GC/cGMP/protein kinase G (PKG) pathway.

Drugs that affect DA homeostasis (synthesis, reuptake, metabolism, or receptors) comprise one of the largest classes of pharmaceuticals. Such drugs are used to treat Parkinson’s disease, schizophrenia, substance abuse, addiction, bipolar disorder, and autism, as well as several peripheral diseases such as hyperprolactinemia, renal hypertension, and GI motility disorders. Drugs that target D2R constitute the largest category of therapeutic dopaminergic agents, and many antipsychotic drugs possess a D2R antagonistic activity. On the other hand, given the high homology in the ligand binding pocket of D1R and D5R, currently available drugs do not discriminate well between these two receptors^[12]^.

Among the DAR-targeting drugs, Fenoldopam (Fen) is especially well suited as a potential anti-cancer drug. Fen is a selective, high affinity (Kd = 2.3 nmol/L), peripheral D1R agonist that does not cross into the brain. Fen is FDA-approved to treat renal hypertensive crisis, and causes only a small drop in diastolic blood pressure in normotensive patients^[13]^. Given its short half-life in circulation, Fen is commonly administered to patients by infusion.

## Non-canonical signaling of the DARs: the cGMP/PKG system

The concept of non-canonical signaling pathways of DARs is based on recent knowledge of the roles played by proximal effectors induced by the receptors such as G-protein-independent β-arrestins and G-protein-coupled receptor kinases, as well as the combinatorial signaling outcome resulting from receptor oligomerization. Among the additional pathways that mediate DAR actions, the cGMP/PKG pathway is of particular interest to this review because it appears to be the major signaling pathway activated by D1R in BC^[2,14]^.

**Figure 3:**
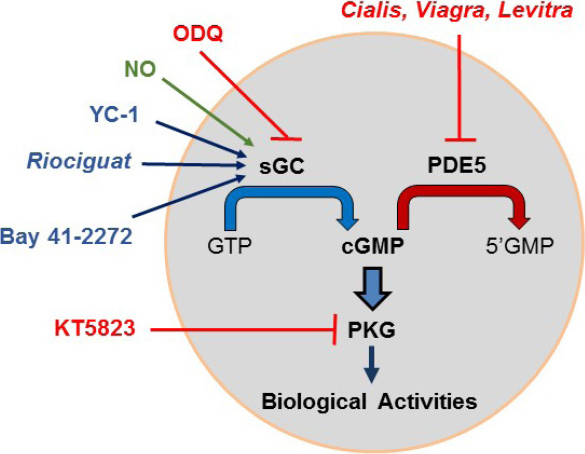
Characteristics of the cGMP/protein kinase G (PKG) pathway. Soluble guanylate cyclase (sGC) is activated by nitric oxide (NO) and by three drugs, namely Riociguate, YC-1, and Bay 41-2271, and is inhibited by ODQ. Activated sGC converts GTP to cGMP while phosphodiesterase 5 (PDE5) hydrolyzes cGMP to 5’GMP. PDE5 inhibitors include tadalafil (Cialis), sildenafil (Viagra) and vardenafil (Levitra). Elevated cGMP activates PKG, which affects many important targets

As illustrated in Figure 3, cGMP is generated from GTP by guanylate cyclase. There are two distinct guanylate cyclases: particulate (pGC) and sGC. The pGCs are transmembrane receptors that primarily bind to natriuretic peptides, while the cytosolic heterodimeric sGC serves as the main target of nitric oxide^[15,16]^. Several drugs, including YC-1, BAY 41-2272, and Riociguat, directly stimulate sGC, while ODQ is a selective inhibitor. Once elevated, the cyclic nucleotides, i.e., cAMP and cGMP, are rapidly hydrolyzed by phosphodiesterases (PDEs), a superfamily of 11 enzymes that differ in structure, catalytic properties, and subcellular localization^[17]^. Based on substrate specificity, the PDEs are grouped into three classes: PDE 4, 7 and 8 selectively hydrolyze cAMP; PDE 5, 6, and 9 are specific for cGMP; and the remainder have dual activity. Several PDE5 inhibitors, e.g., sildenafil (Viagra), tadalafil (Cialis), and vardenafil (Levitra), are widely used to treat erectile dysfunction, acting by causing dilation of penile blood vessels^[18,19]^. Cialis has the longest half-life of the PDE5 inhibitors.

The main targets of cGMP are two kinases, namely PKG-I and PKG-II^[20]^, both of which are inhibited by KT5823. The *PKG-I* gene is expressed as cytosolic PKG-Iα and PKG-Iβ isoforms, while the *PKG-II* gene is expressed as a membrane-associated PKG-II protein. The kinetics, localization, and substrates of the PKG enzymes differ. The PKGs are serine/threonine-specific protein kinases which phosphorylate a number of biologically important targets, some of which overlap with those that are targeted by PKA, while others are distinct. In many, but not all, cell types, activated PKG leads to the suppression of cell proliferation and/or induction of apoptosis.

## Breast cancer attributes

BC is the most common malignancy among women, with more than one million cases occurring annually worldwide, presenting a critical global health challenge. BC primarily arises in the terminal ductal-lobular units, which represent only 10% of the total volume of the mature human breast. Increased risk of developing BC is correlated with early menarche, nulliparity, late age at first childbirth, and late menopause, as well as with obesity and exposure to exogenous hormones (e.g., oral contraceptives and xenoestrogens). Mutations in *BRCA1* and *BRCA2* genes are associated with the increased risk of both breast and ovarian cancers, although heritable breast cancer accounts for no more than 10%-15% of all cases^[21]^. Mutations in *ataxia telangiectasia* are also indicative of increased risk of BC.

BC is classified by different criteria, with the major purpose of classification being to select the best treatment. Historically, patient management decisions have been based on histologic analyses, i.e., tumor size, grade, proliferation indices, lymph node status, and presence of hormone receptors: estrogen (ER), progesterone (PR), and epidermal growth factor receptor 2 (Her2). In recent years, various molecular techniques, particularly gene expression profiling, have been used increasingly to refine the classification of BC and assess prognosis and response to therapy.

Based on the genes expressed by BC, four subtypes are recognized: (1) luminal A is ER^+^, PR^+^, and Her2^-^, with low levels of proliferation index. This is the most common subtype, occurring in about half of the patients. These are low-grade tumors which grow slowly and have the best prognosis; (2) luminal B is ER^+^ and PR^+^, and is either Her2^+^ positive or Her2^-^, but with high levels of proliferation index. These tumors, which occur in about 25% of cases, grow slightly more quickly than luminal A tumors, and their prognosis is slightly worse; (3) Her2-enriched and ER^-^/PR^-^ cancers grow quickly and have a worse prognosis. However, they are often successfully treated with targeted therapies aimed at the Her2 protein; and (4) basal-like, triple-negative tumors are ER^-^, PR^-^, and Her2^-^. This subtype is more common in women with *BRCA1* gene mutations, as well as in younger and African-American women. Most triple-negative tumors are aggressive with a poor prognosis. There are additional refinements of the BC classification, including the status of claudins, and transmembrane proteins that are enriched in tight junctions and are involved in cell migration and epithelial-mesenchymal transition.

The biomedical community is confronted by BC on two fronts: how to implement an early accurate detection and diagnosis, and how to provide an effective clinical management^[22]^. Some tumors are aggressive and life-threatening and must be managed robustly, i.e., by surgery, radiotherapy, and chemotherapy. Neoadjuvant chemotherapy is used to reduce tumor size before surgery, while adjuvant chemotherapy is used after tumor excision. Chemotherapy is the mainstay treatment for patients with triple negative tumors that are resistant to hormone therapy, and for those with advanced metastatic disease^[23]^. Over the years, dozens of anticancer drugs have been developed, with the treatment options taking into account tumor grade and histology and whether the desired outcome is curative or palliative. Most regimens combine drugs that act by different mechanisms so as to improve the odds of suppressing tumor growth.

Endocrine therapy is aimed at suppressing the tumorigenic actions of estrogens and Her2. It has become an established adjuvant therapy, administered after surgery or radiotherapy, but also prior or subsequent to chemotherapy. Effective drugs for ER-positive cancers belong to three main categories: (1) selective ER modulators such as Tamoxifen; (2) aromatase inhibitors such as Anastrozol; and (3) selective ER down-regulators such as Fulverstrant^[24]^. Drugs used to treat Her2-positive tumors belong to two categories: monoclonal antibodies (mAbs) against the receptor, e.g., trastuzumab (Herceptin), and small molecule inhibitors of receptor signaling, e.g., Lapatinib^[25]^. Over time, however, many treated tumors develop resistance to the therapies, curbing the success of treatment.

Preclinical and clinical studies have shown that immunotherapy has the potential to improve outcomes for some patients with triple negative BC. Recently, the FDA approved the first immunotherapy drug, an anti-PD-L1 antibody called Atezolizumab, in combination with chemotherapy, for the treatment of about 20% of triple-negative, metastatic breast tumors that express the PD-L1 protein^[26]^. When functioning properly, activated T lymphocytes can attack tumor cells and curb their growth. However, tumors that express PD-L1 can evade the immune system by inhibiting the anti-tumor activity of effector T cells. The immunotherapy works by blocking and neutralizing PD-L1, enabling the immune system to attack the tumors.

## Overexpression and actions of D1R in breast cancer

DARs are expressed in many peripheral tissues and in some cancers^[9]^. However, previously there was scant information on their expression in BC. A small study in 1986^[27]^ reported binding of [^3^H]spiperone, a D2R-like antagonist, in breast tumors, while a 2012 report identified expression of D3R and D5R in leukemic and BC stem cells^[28]^. Our 2016 report in *Oncogene*^[2]^ was the first to document D1R overexpression in BC, to identify its coupling to the cGMP/PKG apoptotic pathway, and to demonstrate the efficacy of D1R activators in the induction of apoptosis in cultured BCC, and the suppression of BC xenografts in athymic mice. The role of D1R in BC was subsequently supported by others, reporting that D1R activators suppressed BCC migration and bone metastases^[29]^. A 2019 study^[30]^ found that two D1 receptor agonists, Fenoldopam and l-stepholidine, decreased lung metastasis in a 4T1 mouse breast cancer model. A summary of data from our laboratory is presented below.

**Figure 4:**
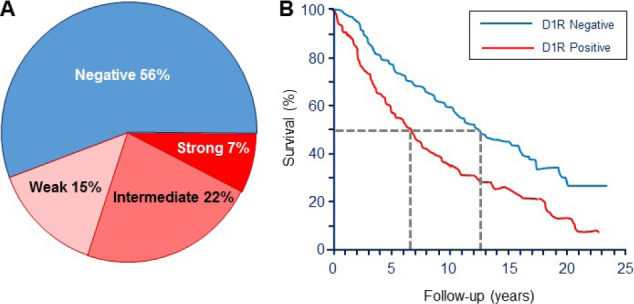
Expression of dopamine receptor-1 (D1R) in breast carcinomas and patient survival. A: Distribution of immunoreactive D1R in tissue microarrays containing 751 breast carcinomas and 30 normal breast samples. Data are shown as the percent of total tumor number. All 30 normal tissue samples were also D1R-negative; B: positive D1R expression in breast tumors is associated with shorter patient survival, as determined by Kaplan-Meier analysis of 508 tumors. Redrawn and modified^[2]^

Using both RT-PCR and immunocytochemistry, we discovered overexpression of D1R in about half of a representative sample of primary breast tumors, but not in adjacent normal breast tissue^[2]^. Subsequently, we used tissue microarrays containing 751 breast tumors and 30 normal breast tissue samples to score D1R expression by immunocytochemistry. As shown in Figure 4A, strong to intermediate D1R staining was evident in ~30% of these tumors, 15% had a weak signal, and the remainder, as well as all normal breast tissue samples, were D1R-negative. Additional analysis of the available information on specimens within the microarrays revealed that D1R staining was significantly associated with pre-menopausal age, and ER^-^, PR^-^, but Her2^+^ tumors. Moreover, D1R-positive tumors were significantly associated with hallmarks of advanced cancer: higher tumor stage, higher tumor grade, and node metastases. Notably, Kaplan-Meyer analysis of 508 tumors with survival data revealed that patients with D1R-positive tumors had a significantly shorter median survival time: 7.5 years *vs*. 12.5 years for those with D1R-negative tumors [Figure 4B]. Based on these findings, we concluded that D1R-overexpressing tumors constitute a high-risk category and do not fit within the conventional “triple negative” category.

**Figure 5:**
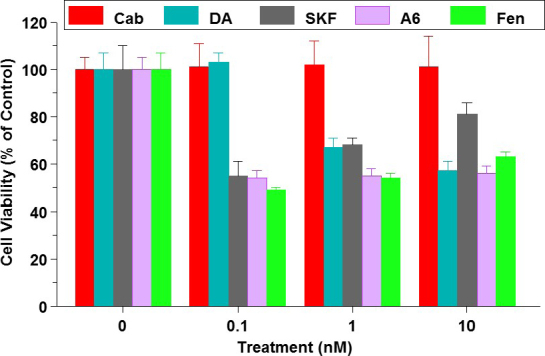
Suppression of MDA-MB-231 cell viability by DA and three dopamine receptor-1 (D1R)-selective agonists. SKF: SKF38393; A6: A68930; Fen: Fenoldopam. Cabergoline (Cab), a D2R agonist, has no effects. Redrawn and modified^[2]^

We also compared D1R and D2R expression and immunoreactivity in eight BCC. The D1R protein was more abundant in aggressive, triple-negative cells than in ER-positive cells. All cells also expressed variable amounts of D2R. Cloning of the *DRD1* transcript from MDA-MB-231 cells confirmed its identity with the published sequence. The putative roles of D1R in BCC were then examined. Based on data obtained with other overexpressed receptors in BC (e.g., ER and EGFR), we fully expected that D1R-agonists would stimulate cell growth. Surprisingly, low nmol/L doses of DA or three D1R-selective agonists, namely SKF38393, A68930 and Fen, but not cabergoline, a D2R agonist, caused a 50% suppression of the viability of MDA-MB-231 [Figure 5]. Similar reductions in cell viability were obtained with other triple negative BCC, namely MDA-MB-468, SUM159, and BT-20, but there were little or no effects of the D1R agonists on ER/PR-positive T47D or MCF7 cells.

Reduced cell viability can be due to lower cell proliferation or to increased apoptosis. After finding that BrdU uptake was not altered by the D1R agonists, we focused on apoptosis. Incubation of MDA-MB-231 or BT-20 cells with DA or SKF38393 increased the percent of apoptotic cells 2-4-fold, as determined by flow cytometry and TUNEL, while 1 nmol/L Fen caused cleavage of caspase 9. Boyden chambers with Matrigel-coated porous membranes were then used to examine if D1R affects cell invasion. DA or Fen was added to cells plated in the upper chambers, while the lower chambers had 10% FBS, which contains chemo-attractants. After 24 h, invaded cells were counted (an insufficient time to reduce cell number by apoptosis). DA or Fen at 1 or 10 nmol/L inhibited FBS-stimulated cell invasion by 70%. To examine if D1R affects chemosensitivity, MDA-MB-468 cells were incubated with doxorubicin, or were pretreated with the D1R agonist A68930 followed by doxorubicin. The results showed that D1R activation increased cell sensitivity to doxorubicin by ~10-fold.

DA has been reported to induce apoptosis in neuroblastoma^[31]^, leukemia^[28]^, ovarian caner^[32]^ and breast cancer^[28,33,34]^. However, most of these studies did not identify which DAR is involved, and DA or its agonists were often used at high pharmacological doses (μmol/L), raising the possibility of non-receptor-mediated toxicity. In contrast, we used molecular cloning and RT-PCR to confirm expression of the *DRD1* gene in primary breast tumors and several BCC. We also used well-validated anti-D1R mAbs to verify expression of the D1R protein in tumors and BCC. Moreover, D1R agonists at low nmol/L doses induced apoptosis, inhibited cell invasion, and increased chemosensitivity^[2]^.

**Figure 6:**
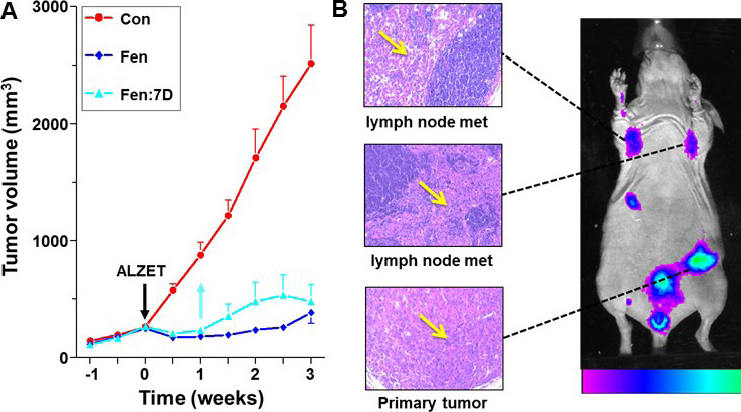
Suppression of xenograft growth by Fenoldopam and fluorescent imaging of dopamine receptor-1 (D1R)-expressing tumors. Treatment with Fenoldopam (Fen), delivered by Alzet osmotic mini-pumps, markedly reduced growth of SUM159-derived xenografts in athymic nude mice (A); Control (Con), no pumps; Fen, mice had the pumps for three weeks; Fen: 7D, mice had the pumps removed after seven days. *n* = 6-8 mice/treatment. Fluorescence imaging of D1R-expressing xenografts (B). Mice with MDA-MB-231-derived tumors were *i.v.* injected with human anti-D1R antibody conjugated to Alexa-Fluor 647. *In vivo* fluorescence imaging after 24 h shows intense fluorescence of the primary tumor and metastases. Arrows in insets show H&E staining of primary tumor and metastases in axillary lymph nodes. Redrawn and modified^[2]^

We next examined the effects of D1R activation on growth of xenografts. Athymic nude mice were orthotopically implanted with either of two highly tumorigenic BCC: MDA-MB-231 and SUM159. When tumor volume reached ~150 mm^3^, mice were treated with Fen, delivered by subcutaneously-implanted Alzet osmotic mini-pumps, rated for continuous delivery of their cargo at 0.11 μL/h for four weeks. As evident in Figure 6A, the D1R agonist caused a dramatic suppression of tumor growth. Moreover, the suppression of tumor growth by Fen was long lasting, as treated tumors remained quiescent for at least two additional weeks after removal of the Alzet pumps (Figure 6A, light blue line and arrow). After three weeks, tumors were resected and analyzed. Tumor reduction to an almost undetectable size was due to increases in both apoptosis and necrosis. The combination of apoptosis and necrosis likely explains the more robust suppressive effects of Fen *in vivo* than *in vitro*. Increased necrosis could have been due to the inhibition of angiogenesis, since DA was reported to reduce tumor angiogenesis by inhibiting VEGF^[35]^, and its receptor^[36]^ via endothelial DARs^[32]^.

A fluorescent imaging method for visualizing D1R-expressing tumors and metastases was also developed. Figure 6B shows intense fluorescence of primary tumors and axillary metastases in mice with MDA-MB-231-derived xenografts, which were intravenously injected (*i.v.*) with human anti-D1R antibody conjugated to Alexa-Fluor 647. Although fluorescence imaging is suitable for detecting superficial tumors and metastases in mice, given the limited penetration of fluorescence signals, this approach is inappropriate for imaging deep-seated tumors or metastases in humans. Instead, we have begun to develop PET (positron emission tomography) imaging using TISCH (7-chloro-8-hydroxy-1-(3’-iodophenyl)-3-methyl-2,3,4,5-tetrahydro-1H-3-benzazepine), an iodine-containing benzazepine. TISCH is the only ligand with high selectivity for D1R that is suitable for PET^[37]^. When labeled with ^125^I, TISCH retained high binding affinity to D1R and selectively tagged the rat striatum and cerebral cortex^[38]^.

## Activation of the cGMP/PKG system in breast cancer

**Figure 7:**
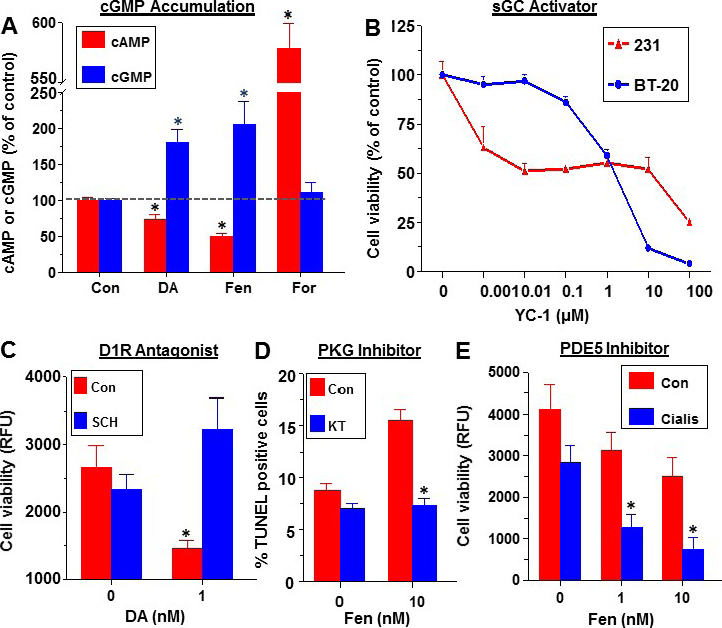
Evidence for activation of the cGMP/protein kinase G (PKG) axis by dopamine receptor-1 (D1R) in breast cancer. Increased accumulation of cGMP in MDA-MB-231 cells by DA and Fenoldopam (Fen) and inhibition of cAMP. For: forskolin (A); suppression of cell viability in MDA-MB-231 and BT-20 cells by YC-1, an activator of soluble guanylate cyclase (B); abrogation of DA-induced suppression of MDA-MB-468 cell viability by pre-incubation with a D1R antagonist SCH39166. RFU: relative fluorescence units (C); preincubation with PKG inhibitor KT5823 prevents the induction of apoptosis in SUM159 cells by Fenoldopam (D); co-incubation of Cialis, an antagonist of PDE5, with Fen augments the suppression of cell viability in SUM159 cells by Fen alone (E). Redrawn and modified^[2]^. *Designates significant (P < 0.05) over control

Since D1R agonists are classified as cAMP activators, the effect of D1R activation on cAMP accumulation was examined. Unexpectedly, short-term incubation of MDA-MB-231 cells with DA or Fen significantly increased intracellular cGMP levels while suppressing cAMP [Figure 7A]. The marked increase in cAMP in response to forskolin, a direct AC activator, indicated that these cells have an intact AC/cAMP machinery. The decrease in cAMP in response to DA or Fen is likely secondary to the elevated cGMP, which activates cAMP-hydrolyzing PDEs, underlying the reciprocal relationships between the two cyclic nucleotides^[39]^.

The involvement of the cGMP/PKG axis was verified by conducting several complementary experiments. First, the direct stimulation of sGC by YC-1 in MDA-MB-231 and BT-20 caused a dose-dependent decrease in cell viability [Figure 7B]. Second, the authenticity of D1R activation by DA was confirmed by abrogating the DA-induced decrease in MDA-MB-468 cell viability by pre-incubation with SCH39166 (SCH), a D1R antagonist [Figure 7C]. Third, KT5823, a selective PKG inhibitor, prevented the Fen-induced apoptosis in SUM159 cells [Figure 7D]. Fourth, the blockade of PDE5 by Cialis (tadalafil) increased cGMP levels, induced PKG activity, and augmented the Fen-induced apoptosis in SUM159 cells [Figure 7E].

Our observations were supported by the report that cGMP, via PKG activation, suppressed growth of both ER-positive and ER-negative BCC^[40]^. In addition, sildenafil (Viagra) was reported to increase cytotoxicity by doxorubicin, cisplatin, and paclitaxel, and its co-administration with doxorubicin enhanced the suppression of xenograft growth^[41]^. More recently, cGMP in breast tumors in mice was significantly elevated by Fen, while cAMP was only mildly elevated^[30]^.

The ubiquitous cAMP/PKA pathway has been extensively studied, while knowledge of the less common cGMP/PKG pathway lags behind, in part because of conflicting reports on its effects on apoptosis^[20]^. Some breast^[40,42]^ and colon^[43,44]^ tumors have reduced PKG or elevated PDE5 expression. These appear to confer growth advantage to these tumors, since activation of the cGMP/PKG axis, PKG overexpression, or PDE5 downregulation in these cells inhibit their growth. Given the many targets of PKGs, there is no universal mechanism by which they induce apoptosis. For example, in MCF7 and MDA-MB-468 cells, YC-1 increased cGMP, arrested the cell cycle, and activated caspase 9^[40]^. Using the same cells, others reported that a pharmacological suppression or the downregulation of PDE5 induced apoptosis by attenuating the oncogenic Wnt/βCatenin pathway^[42]^.

Inhibitors of PDE5 enhance drug sensitivity in several cancers^[45]^. For instance, treatment of prostate cancer cells with Sildenafil augmented doxorubicin-induced apoptosis, activated caspases, and reduced Bcl-xL expression, while co-treatment of prostate tumor xenografts with Sildenafil and doxorubicin enhanced the suppression of tumor growth^[46]^. In medulloblastoma^[47]^ and gastrointestinal cancer^[48]^, a combination of PDE5 inhibitors and anti-cancer drugs was more effective than each alone in enhancing cell death. In BCC, Sildenafil increased the efficacy of cell killing by doxorubicin, cisplatin, and paclitaxel, and its co-administration with doxorubicin enhanced the suppression of xenograft growth^[41]^. Several clinical trials are currently testing the efficacy of PDE5 inhibitors, alone or in combination with chemotherapeutic agents, in prostate, head and neck, and pancreatic cancers^[49]^.

## Conclusions, perspectives, and relevance to patients

**Figure 8:**
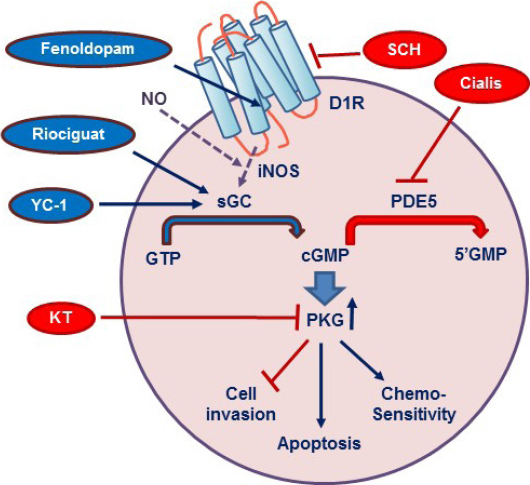
A model of the interactions of dopamine receptor-1 (D1R) with the cGMP/protein kinase G (PKG) system. The association of D1R with inducible nitric oxide synthase (iNOS) is assumed but not proven. See text for other explanations

Figure 8 illustrates the major players involved with the D1R-cGMP-PKG system in breast cancer. Binding of D1R agonists to the receptor increases intracellular cGMP levels by activating soluble guanylate cyclase. We postulate that D1R is linked to iNOS via the heterotrimeric Gα12 or Gα13 subunits, reported to be coupled to iNOS^[50,51]^. In preliminary studies, we found that Gα12 expression was 8- and 30-fold higher in MDA-MB-468 and SUM159 cells, respectively, than in the human heart; they also had high expression of iNOS. Elevated cGMP activates PKG, which in turns suppresses cell invasion, stimulates apoptosis, and increases chemosensitivity. A number of drugs, some of which are FDA-approved, can cause the same effects. For example, Riociguat (Adempas), a second generation YC-1, which stimulates guanylate cyclase, is available in a tablet form to treat chronic pulmonary hypertension. Cialis (tadalafil), which increases cGMP by inhibiting PDE5, is currently in phase I/II clinical trials in patients with head and neck cancer^[52]^. In addition to its ability to suppress tumor growth, Cialis boosts the capacity of the immune system to eliminate cancer cells^[53]^.

We offer a novel concept that D1R can serve as a biomarker for prognosis in advanced BC, and its agonists can be used as effective therapeutics. Among dozens of agonists and antagonists with high selectivity for DAR subtypes, only few, exemplified by Fen, do not cross the blood-brain barrier and target only peripheral receptors. Fen is a small molecule with a mass of 305 g/mol. Small molecules have proven highly valuable for treating many diseases, and most oral medicines marketed today belong to this class. Although Fen is currently delivered to patients with renal hypertension by *i.v.* infusion, pharmaceutical companies commonly devise slow release formulation of many drugs with prolonged activity and oral deliverability.

Identification of patients who could benefit from D1R-targeted therapy is of critical importance. This can be achieved by two methods: (1) analysis of D1R in tumor biopsies by immuno-histochemistry, as is currently done for ER and Her2/neu; and (2) non-invasive imaging. Medical imaging modalities are designed to diagnose, stage, and evaluate the response to therapy in BC^[54]^. PET uses positron-emitting radioisotopes to image tumors with high sensitivity and excellent resolution. PET is based on the functional readouts of tumor properties such as high metabolism, and expression of unique receptors, with the ultimate goal of identifying effective therapeutic targets^[55]^. Advantages of PET include a non-invasive assessment of metastases that are not accessible to biopsy, and a serial monitoring of drug effects. The use of PET imaging has become more widespread in the detection and treatment of patients with BC^[56]^. Nonetheless, many clinical studies will be needed before D1R can be established as a prognosis biomarker.

## Unresolved questions and potential future studies

It is counter-intuitive that increased D1R expression correlates with advanced BC and shorter patient survival, while D1R activation, rather than its suppression, causes apoptosis. This enigma raises several questions: (1) what is the molecular mechanism responsible for the higher expression of D1R in tumors than in normal tissue? (2) what is the linkage between D1R and the sGC/PKG/apoptotic pathway, and is this linkage disrupted in advanced BC but can be restored upon exposure to a potent D1R agonist? (3) does the *DRD1* gene undergo some transformations during tumorigenesis that result in a non-conventional association with an oncogenic pathway(s)? (4) do certain D1R-expressing tumors in patients continue to grow and do not undergo apoptosis in response to circulating DA-S because they lack ARSA expression, and/or because they overexpress D2R-like which antagonize D1R-induced apoptosis?

Instead of increasing cAMP levels, as is the case in most tissues, D1R agonists increase cGMP levels in BCC. It is possible that the switch in the secondary messenger and the signaling pathway is the consequence of the process of tumorigenesis. Future studies could compare the responsiveness of BCC to D1R agonists with those of non-malignant breast epithelial cell lines as well as of cells isolated from early tumors and normal adjacent tissues.

Future studies should also analyze expression of Gαs, Gαq, Gα12, and Gα13, as well as all NOS isoforms, followed by immunoblotting. Physical association between D1R and any identified G protein could be examined by co-immunoprecipitation, using formaldehyde for reversible cross-linking, followed by pulldown and blotting. Functional links between D1R and Gα12 or Gα13 could also be verified by knocking out each identified G protein, using a CRISPR/Cas 9 approach, as well as by a transient transfection with a dominant negative mutant, e.g., Gα13Q226L^[50]^, followed by analysis of Fen-induced iNOS activity, or its effects on cGMP accumulation.

D1R may have a ligand-independent action in BC (as is the case with Her2), and if so, it would promote tumor growth, but could be directed toward the apoptotic pathway upon exposure to a potent agonist. Ligand-independent receptor action, displayed as a constitutive activity, is a rather common phenomenon in hormone-sensitive cancers, and often results from mutations in a critical domain of the receptor. Indeed, residue Ile288, located in the third cytoplasmic loop of D5R (which links the receptor to G-proteins), is associated with constitutive activity, and was silenced when substituted with Phe^[57]^. Next generation sequencing of the *DRD1* gene in primary tumors could identify mutated receptors. If so, the mutated receptor could be cloned and expressed in Hek293 cells^[58]^, or in Fen-unresponsive BCC, followed by analysis of cGMP levels and apoptosis with and without treatment with D1R agonists and antagonists.

It is possible that D1R does not have to be overexpressed in order to respond to a given ligand, but low to intermediate levels of expression suffice. If so, this raises the questions whether circulating DA exerts stimulatory, inhibitory, or no effects on D1R-expressing tumors, and whether the type of response depends upon serum DA-S levels, tumor ARSA activity, and/or the balance between D1-like and D2-like receptor expression in any given tumor. Unfortunately, the presence of DA-S in human serum has been overlooked by most investigators, since DA-S is undetectable by most routine analytical methods for catecholamines and requires a special extraction method. We speculate that serum DA-S suppresses the growth of D1R expressing tumors only if they have both: a functional and releasable ARSA, and low expression of D2-like receptor. Both serum DA-S and ARSA levels could be used as future predictors of tumor development and responsiveness to D1R agonists.

Another issue that deserves attention is whether a prolonged exposure to D1R agonists desensitize the cells to the ligand, as is the case for adrenergic receptors^[59]^, and some DARs^[60]^, and whether intermittent, repeated exposures to Fen are preferred over continuous exposure. This can be experimentally resolved by comparting the effects of continuous *vs*. interrupted Fen delivery under both *in vitro* and *in vivo* conditions.

Another question is whether systemic metastases of BC maintain the same D1R status (negative or positive) as do primary tumors. Nonetheless, D1R expression by metastases *per se* does not necessarily predict an apoptotic response to D1R agonists in patients. Other biomarkers, e.g., iNOS and sGC, may be required. Moreover, drugs such as Riociguat and Cialis, which bypass D1R altogether, could be effective in tumors that have no, low, or non-functional D1R. Finally, tumors other than the breast (e.g., in the colon, prostate, or ovary) may also overexpress D1R, which will greatly expand the scope of future studies on the role of DARs in tumorigenesis.

## Declarations

### Copyright

© The Author(s) 2019.
